# Approximation of Bivariate Functions via Smooth Extensions

**DOI:** 10.1155/2014/102062

**Published:** 2014-02-10

**Authors:** Zhihua Zhang

**Affiliations:** College of Global Change and Earth System Science, Beijing Normal University, Beijing 100875, China

## Abstract

For a smooth bivariate function defined on a general domain with arbitrary shape, it is difficult to do Fourier approximation or wavelet approximation. In order to solve these problems, in this paper, we give an extension of the bivariate function on a general domain with arbitrary shape to a smooth, periodic function in the whole space or to a smooth, compactly supported function in the whole space. These smooth extensions have simple and clear representations which are determined by this bivariate function and some polynomials. After that, we expand the smooth, periodic function into a Fourier series or a periodic wavelet series or we expand the smooth, compactly supported function into a wavelet series. Since our extensions are smooth, the obtained Fourier coefficients or wavelet coefficients decay very fast. Since our extension tools are polynomials, the moment theorem shows that a lot of wavelet coefficients vanish. From this, with the help of well-known approximation theorems, using our extension methods, the Fourier approximation and the wavelet approximation of the bivariate function on the general domain with small error are obtained.

## 1. Introduction

In the recent several decades, various approximation tools have been widely developed [1–14]. For example, a smooth periodic function can be approximated by trigonometric polynomials; a square-integrable smooth function can be expanded into a wavelet series and be approximated by partial sum of the wavelet series; and a smooth function on a cube can be approximated well by polynomials. However, for a smooth function on a general domain with arbitrary shape, even if it is infinitely many time differentiable, it is difficult to do Fourier approximation or wavelet approximation. In this paper, we will extend a function on general domain with arbitrary shape to a smooth, periodic function in the whole space or to a smooth, compactly supported function in the whole space. After that, it will be easy to do Fourier approximation or wavelet approximation. For the higher-dimensional case, the method of smooth extensions is similar to that in the two-dimensional case, but the representations of smooth extensions will be too complicated. Therefore, in this paper, we mainly consider the smooth extension of a bivariate function on a planar domain. By the way, for the one-dimensional case, since the bounded domain is reduced to a closed interval, the smooth extension can be regarded as a corollary of the two-dimensional case.

This paper is organized as follows. In Section 2, we state the main theorems on the smooth extension of the function on the general domain and their applications. In Sections 3 and 4, we give a general method of smooth extensions and complete the proofs of the main theorems. In Section 5, we use our extension method to discuss two important special cases of smooth extensions.

Throughout this paper, we denote *T* = [0,1]^2^ and the interior of *T* by *T* ^*o*^ and always assume that *Ω* is a simply connected domain. We say that *f* ∈ *C* ^*q*^(*Ω*) if the derivatives (∂^*i*+*j*^ *f*/∂*x* ^*i*^∂*y* ^*j*^) are continuous on *Ω* for 0 ≤ *i* + *j* ≤ *q*. We say that *f* ∈ *C* ^*∞*^(*Ω*) if all derivatives (∂^*i*+*j*^ *f*/∂*x* ^*i*^∂*y* ^*j*^) are continuous on *Ω* for *i*, *j* ∈ *ℤ*_+_. We say that a function *h*(*x*, *y*) is a *γ*-periodic function if *h*(*x* + *γk*, *y* + *γl*) = *h*(*x*, *y*)  ((*x*, *y*) ∈ *T*; *k*, *l* ∈ *ℤ*), where *γ* is an integer. We appoint that 0! = 1 and the notation [*α*] is the integral part of the real number *α*.

## 2. Main Theorems and Applications

In this section, we state the main results of smooth extensions and their applications in Fourier analysis and wavelet analysis.

### 2.1. Main Theorems

Our main theorems are stated as follows.

**Theorem 1**

Let *f* ∈ *C* ^*∞*^(*Ω*), where *Ω* ⊂ *T* ^*o*^ and the boundary ∂*Ω* is a piecewise infinitely many time smooth curve. Then for any *r* ∈ *ℤ*_+_ there is a function *F* ∈ *C* ^*r*^(*T*) such that

(i) *F*(*x*, *y*) = *f*(*x*, *y*)  ((*x*, *y*) ∈ *Ω*);
(ii) (∂^*i*+*j*^ *F*/∂*x* ^*i*^∂*y* ^*j*^)(*x*, *y*) = 0 on the boundary ∂*T* for 0 ≤ *i* + *j* ≤ *r*;
(iii) on the complement *T*∖*Ω*, *F*(*x*, *y*) can be expressed locally in the forms
  (1) $$\begin{array}{c} \overset{L}{\underset{j=0}{\sum }}\;\xi_{j}\left(x\right)y^{j}‍,\;or\;\overset{L}{\underset{j=0}{\sum }}\eta_{j}\left(y\right)x^{j}‍,\;or\;\overset{L}{\underset{i,j=0}{\sum }}c_{ij}x^{i}y^{j}‍, \end{array}$$

where *L* is a positive integer and each coefficient *c*_*ij*_ is constant.

**Theorem 2**

Let *f* ∈ *C* ^*∞*^(*Ω*), where *Ω* is stated as in Theorem 1. Then, for any *r* ∈ *ℤ*_+_, there exists a 1-periodic function *F*_*p*_ ∈ *C* ^*r*^(ℝ^2^) such that *F*_*p*_(*x*, *y*) = *f*(*x*, *y*)  ((*x*, *y*) ∈ *Ω*).

**Theorem 3**

Let *f* ∈ *C* ^*∞*^(*Ω*), where *Ω* is stated as in Theorem 1. Then, for any *r* ∈ *ℤ*_+_, there exists a function *F* ^*c*^ ∈ *C* ^*r*^(ℝ^2^) with compact support *T* such that *F* ^*c*^(*x*, *y*) = *f*(*x*, *y*)  ((*x*, *y*) ∈ *Ω*).

In Sections 3 and 4, we give constructive proofs of Theorems 1–3. In these three theorems, we assume that *f* ∈ *C* ^*∞*^(*Ω*). If *f* ∈ *C* ^*q*^(*Ω*) (*q* is a nonnegative integer), by using the similar method of arguments of Theorems 1–3, we also can obtain the corresponding results.

### 2.2. Applications

Here we show some applications of these theorems.

#### 2.2.1. Approximation by Polynomials

Let *F* be the smooth extension of *f* from *Ω* to *T* which is stated as in Theorem 1. Then *F* ∈ *C* ^*r*^(*T*) and *F* = *f* on *Ω*. By Δ_*N*_, denote the set of all bivariate polynomials in the form ∑_*n*_1_,*n*_2_=−*N*_^*N*^ *c*_*n*_1_,*n*_2__ *x* ^*n*_1_^ *y* ^*n*_2_^. Then

(2) $$\begin{array}{c} \underset{P\in \Delta_{N}}{\inf}{||f-P||}_{L_{p}(\Omega )}\leq \underset{P\in \Delta_{N}}{\inf}{||F-P||}_{L_{p}(T)}, \end{array}$$

where ||·||_*L*_*p*_(*D*)_ is the norm of the space *L*_*p*_(*D*). The right-hand side of formula (2) is the best approximation of the extension *F* in Δ_*N*_. By (2), we know that the approximation problem of *f* by polynomials on a domain *Ω* is reduced to the well-known approximation problem of its smooth extension *F* by polynomials on the square *T* [4, 10].

#### 2.2.2. Fourier Analysis

(i) *Approximation by Trigonometric Polynomials.* Let *F*_*p*_ be the smooth periodic extension of *f* as in Theorem 2. Then *F*_*p*_ ∈ *C* ^*r*^(ℝ^2^) and *F*_*p*_ = *f* on *Ω*. By the well-known results [5, 10], we know that the smooth periodic function *F*_*p*_ can be approximated by bivariate trigonometric polynomials very well. Its approximation error can be estimated by the modulus of continuity of its *r* time derivatives.

By Δ_N_*, denote the set of all bivariate trigonometric polynomials in the form

(3) $$\begin{array}{c} \overset{N}{\underset{n_{1},n_{2}=-N}{\sum }}c_{n_{1},n_{2}}^{*}e^{2\pi i(n_{1}x+n_{2}y)}. \end{array}$$

By Theorem 2, we have

(4) $$\begin{array}{c} {\underset{P^{*}\in \Delta_{N}^{*}}{\inf}||f-P^{*}||}_{L_{p^{\prime }}(\Omega )}\leq {\underset{P^{*}\in \Delta_{N}^{*}}{\min}||F_{p}-P^{*}||}_{L_{p^{\prime }}(T)}. \end{array}$$

From this and Theorem 2, we see that the approximation problem of *f* on *Ω* by trigonometric polynomials is reduced to a well-known approximation problem of smooth periodic functions [5, 7, 10].

(ii) *Fourier Series*. We expand *F*_*p*_ into a Fourier series [9]

(5) $$\begin{array}{c} F_{p}\left(x,y\right)=\underset{\left(n_{1},n_{2}\right)\in {\mathbb{Z}}^{2}}{\sum }\tau_{n_{1},n_{2}}e^{2\pi i(n_{1}x+n_{2}y)}, \end{array}$$

where *τ*_*n*_1_,*n*_2__ = ∫_*T*_ *F*_*p*_(*x*, *y*)*e* ^−2*πi*(*n*_1_*x*+*n*_2_*y*)^ *dx* *dy*. By Theorem 2, we obtain that, for (*x*, *y*) ∈ *Ω*,

(6) $$\begin{array}{c} f\left(x,y\right)=\underset{\left(n_{1},n_{2}\right)\in {\mathbb{Z}}^{2}}{\sum }\tau_{n_{1},n_{2}}e^{2\pi i(n_{1}x+n_{2}y)}. \end{array}$$

Denote the partial sum

(7) $$\begin{array}{c} s_{n_{1},n_{2}}\left(x,y\right)=\overset{n_{1}}{\underset{k_{1}=0\;}{\sum }}\overset{n_{2}}{\underset{k_{2}=0}{\sum }}\tau_{k_{1},k_{2}}e^{2\pi i(k_{1}x+k_{2}y)}. \end{array}$$

Then we have

(8) ||f(x,y)−sn1,n2(x,y)||Lp′(Ω) ≤||Fp(x,y)−sn1,n2(x,y)||Lp′(T).

Since the smooth periodic function *F*_*p*_ can be approximated well by the partial sum of its Fourier series [5, 7, 10], from this inequality, we see that we have constructed a trigonometric polynomial *s*_*n*_1_,*n*_2__(*x*, *y*) which can approximate to *f* on *Ω* very well.

(iii) *Odd (Even) Periodic Extension.* Let *F* be the smooth extension of *f* from *Ω* to *T* which is stated in Theorem 1. Define *F* ^*o*^ on [−1,1]^2^ by

(9) $$\begin{array}{c} F^{o}\left(x,y\right)=\{\begin{array}{cc} F\left(x,y\right), & \left(x,y\right)\in {\left[0,1\right]}^{2},\\ -F\left(-x,y\right), & \left(x,y\right)\in \left[-1,0\right]\times \left[0,1\right],\\ F\left(-x,-y\right), & \left(x,y\right)\in {\left[-1,0\right]}^{2},\\ -F\left(x,-y\right), & \left(x,y\right)\in \left[0,1\right]\times \left[-1,0\right]. \end{array} \end{array}$$

Then *F* ^*o*^ is an odd function. By Theorem 1, we have *F* ^*o*^ ∈ *C* ^*r*^([−1,1]^2^) and (∂^*i*+*j*^ *F* ^*o*^/∂*x* ^*i*^∂*y* ^*j*^)(*x*, *y*) = 0 on ∂([−1,1]^2^) for 0 ≤ *i* + *j* ≤ *r*. Again, doing a 2-periodic extension, we obtain a 2-periodic odd function *F*_*p*_^*o*^ and *F*_*p*_^*o*^ ∈ *C* ^*r*^(ℝ^2^). By the well-known results [5, 7, 10], *F*_*p*_^*o*^ can be approximated by sine polynomials very well. Moreover, *F*_*p*_^*o*^ can be expanded into the Fourier sine series; that is,

(10) $$\begin{array}{c} F_{p}^{o}\left(x,y\right)=\overset{\infty }{\underset{n_{1}=1}{\sum }}\;\overset{\infty }{\underset{n_{2}=1}{\sum }}\alpha_{n_{1},n_{2}}\sin\left(\pi n_{1}x\right)\sin\left(\pi n_{2}y\right), \end{array}$$

where the coefficients *α*_*n*_1_,*n*_2__ = 4∫_*T*_ *F*_*p*_^*o*^(*x*, *y*)sin(*πn*_1_ *x*)sin(*πn*_2_ *y*) *dx* *dy* [9]. Considering the approximation of *F*_*p*_^*o*^ by the partial sum, the Fejer sum, and the Vallee-Poussin sum [7, 14] of the Fourier sine series of *F*_*p*_^*o*^, we will obtain the approximation of the original function *f* on *Ω* by sine polynomials.

Define *F* ^*e*^ on [−1,1]^2^ as follows:

(11) $$\begin{array}{c} F^{e}\left(x,y\right)=\{\begin{array}{cc} F\left(x,y\right), & \left(x,y\right)\in {\left[0,1\right]}^{2},\\ F\left(-x,y\right), & \left(x,y\right)\in \left[-1,0\right]\times \left[0,1\right],\\ F\left(-x,-y\right), & \left(x,y\right)\in {\left[-1,0\right]}^{2},\\ F\left(x,-y\right), & \left(x,y\right)\in \left[0,1\right]\times \left[-1,0\right]. \end{array} \end{array}$$

Then *F* ^*e*^ is an even function on [−1,1]^2^. By Theorem 1, *F* ^*e*^ ∈ *C* ^*r*^([−1,1]^2^) and (∂^*i*+*j*^ *F* ^*e*^/∂*x* ^*i*^  ∂*y* ^*j*^)(*x*, *y*) = 0 on ∂([−1,1]^2^) for 0 ≤ *i* + *j* ≤ *r*. Again, doing a 2-periodic extension, we obtain a 2-periodic even function *F*_*p*_^*e*^ and *F*_*p*_^*e*^ ∈ *C* ^*r*^(ℝ^2^). By the well-known result [5, 10], *F*_*p*_^*e*^ can be approximated by cosine polynomials very well. Moreover, *F*_*p*_^*e*^ can be expanded into the Fourier cosine series. Considering the partial sum, the Fejer sum, and the Vallee-Poussin sum [5, 7, 14] of the Fourier cosine series of *F*_*p*_^*e*^, we will obtain the approximation of the original function *f* on *Ω* by cosine polynomials.

#### 2.2.3. Wavelet Analysis

(i) *Periodic Wavelet Series.* Let *F*_*p*_ ∈ *C* ^*r*^(ℝ^2^) be stated in Theorem 2. Let {*ψ*_*μ*_}_1_^3^ be a bivariate smooth wavelet [2]. Then, under a mild condition, the families

(12) Ψper:={1}⋃{ψμ,m,nper,μ=1,2,3;  m∈ℤ+;  n=(n1,n2),n1,n2=0,…,2m−1},where  ψμ,m,nper=∑l∈ℤ2ψμ,m,n(·+l),ψμ,m,n=2mψμ(2m·−n)

are a periodic wavelet basis. We expand *F*_*p*_ into a periodic wavelet series [2]

(13) $$\begin{array}{c} F_{p}=d_{0,0}+\overset{3}{\underset{\mu =1}{\sum }}\overset{\infty }{\underset{\;m=0}{\sum }}\overset{2^{m}-1}{\underset{\;n_{1},n_{2}=0}{\sum }}d_{\mu ,m,n}\psi_{\mu ,m,n}^{\text{per}}. \end{array}$$

From this, we can realize the wavelet approximation of *f* on *Ω*, for example, if *r* = 2, its partial sum

(14) $$\begin{array}{c} s_{2^{M}}\left(F_{p}\right)=d_{0,0}+\overset{3}{\underset{\mu =1}{\sum }}\overset{M-1}{\underset{\;m=0}{\sum }}\overset{2^{m}-1}{\underset{\;n_{1},n_{2}=0}{\sum }}d_{\mu ,m,n}\psi_{\mu ,m,n}^{\text{per}} \end{array}$$

satisfies ||*F*_*p*_−*s*_2^*M*^_(*F*_*p*_)||_*L*^2^(*T*)_ = *O*(2^−2*M*^). From this and *F*_*p*_(*x*, *y*) = *f*(*x*, *y*)  ((*x*, *y*) ∈ *Ω*), we will obtain an estimate of wavelet approximation for a smooth function *f* on the domain *Ω*.

(ii) *Wavelet Approximation*. Let *F* ^*c*^ be the smooth function with a compact support as in Theorem 3. Let *ψ* be a univariate Daubechies wavelet and *ϕ* be the corresponding scaling function [2]. Denoting

(15) ψ1(x,y)=ϕ(x)ψ(y),  ψ2(x,y)=ψ(x)ϕ(y),  ψ3(x,y)=ψ(x)ψ(y),

then {*ψ*_*μ*_(*x*, *y*)}_*μ*=1_^3^ is a smooth tensor product wavelet. We expand *F* ^*c*^ into the wavelet series

(16) $$\begin{array}{c} F^{c}\left(x,y\right)=\overset{3}{\underset{\mu =1}{\sum }}\underset{\;m\in \mathbb{Z}}{\sum }\underset{\;n\in {\mathbb{Z}}^{2}}{\sum }c_{\mu ,m,n}\psi_{\mu ,m,n}\left(x,y\right), \end{array}$$

where *ψ*_*μ*,*m*,*n*_ = 2^*m*^ *ψ*_*μ*_(2^*m*^ · −*n*) and the wavelet coefficients

(17) cμ,m,n=∫ℝ2Fc(x,y)ψ¯μ,m,n(x,y)dx dy  =∫TF(x,y)ψ¯μ,m,n(x,y)dx dy.‍

Since *F* ^*c*^ is a smooth function, the wavelet coefficients *c*_*μ*,*m*,*n*_ decay fast.

On the other hand, since *F* ^*c*^(*x*, *y*) = 0, (*x*, *y*) ∈ ℝ^2^∖*T*, a lot of wavelet coefficients vanish. In fact, when *m*_0_ ∈ *ℤ* and *n*_0_ ∈ *ℤ* ^2^ satisfy supp⁡*ψ*_*μ*,*m*_0_,*n*_0__ ⊂ (ℝ^2^∖*T*), we have *c*_*μ*,*m*_0_,*n*_0__ = 0. Besides, by condition (iii) in Theorem 1, we know that *F* is univariate or bivariate polynomials on *T*∖*Ω*. By the moment theorem [2], we know that more wavelet coefficients vanish.

For example, let m* ∈ *ℤ* and n* = (*n*_1_*, *n*_2_*) ∈ *ℤ* ^2^ satisfy supp⁡*ψ*_m*,*n*_2_*_ ⊂ [0, α*], where α* = inf⁡{*g*(*x*), *x*_1_ ≤ *x* ≤ *x*_2_}. Then we have

(18) c1,m∗,n∗=2m(∫0x1‍+∫x1x2‍+∫x21‍)ϕ¯m∗,n1∗(x)×(∫0α∗ψ¯m∗,n2∗(y)F(x,y)dy)dx  =  :I1+I2+I3.

By Lemma 8, we know that

(19) $$\begin{array}{c} F\left(x,y\right)=\overset{L}{\underset{j=0}{\sum }}\xi_{j}\left(x\right)y^{j},\;\left(x,y\right)\in E_{1}, \end{array}$$

where *E*_1_ = {(*x*, *y*) : *x*_1_ ≤ *x* ≤ *x*_2_, 0 ≤ *y* ≤ *g*(*x*)} and *g*(*x*) ≥ α*(*x*_1_ ≤ *x* ≤ *x*_2_). So

(20) $$\begin{array}{c} I_{2}=\overset{x_{2}}{\underset{x_{1}}{\int }}{\bar{\phi }}_{m^{*},n_{1}^{*}}\left(x\right)\left(\overset{\alpha^{*}}{\underset{0}{\int }}\;{\bar{\psi }}_{m^{*},n_{2}^{*}}\left(y\right)\left(\overset{L}{\underset{j=0}{\sum }}\xi_{j}\left(x\right)y^{j}\right)dy‍\right)dx. \end{array}$$

If the Daubechies wavelet *ψ* chosen by us is *L* time smooth, then, by using the moment theorem and supp⁡*ψ*_m*,*n*_2_*_ ⊂ [0, α*], we have

(21) ∫0α∗ψ¯m∗,n2∗(y)yjdy‍=∫ℝψ¯m∗,n2∗(y)yjdy=0,(0≤j≤2r+1).

So *I*_2_ = 0. Similarly, since *F*(*x*, *y*) is bivariate polynomials on rectangles *H*_1_ and *H*_3_ (see Lemma 11), we have *I*_1_ = *I*_3_ = 0. Furthermore, by (18), we get *c*_1,m*,n*_ = 0.

Therefore, the partial sum of the wavelet series (16) can approximate to *F* ^*c*^ very well and few wavelet coefficients can reconstruct *F* ^*c*^. Since *F* ^*c*^ = *f* on *Ω*, the partial sum of the wavelet series (16) can approximate to the original function *f* on the domain *Ω* very well.

## 3. Proofs of the Main Theorems

We first give a partition of the complement *T*∖*Ω*.

### 3.1. Partition of the Complement of the Domain *Ω* in *T*

Since *Ω* ⊂ *T* ^*o*^ and ∂*Ω* is a piecewise infinitely many time smooth curve, without loss of generality, we can divide the complement *T*∖*Ω* into some rectangles and some trapezoids with a curved side. For convenience of representation, we assume that we can choose four point (*x*_*ν*_, *y*_*ν*_)∈∂*Ω*  (*ν* = 1,2, 3,4) such that *T*∖*Ω* can be divided into the four rectangles

(22) $$\begin{array}{c} H_{1}=\left[0,x_{1}\right]\times \left[0,y_{1}\right],\;\;H_{2}=\left[x_{2,}1\right]\times \left[0,y_{2}\right],\\ H_{3}=\left[x_{3,}1\right]\times \left[y_{3,}1\right],\;\;H_{4}=\left[0,x_{4}\right]\times \left[y_{4,}1\right] \end{array}$$

and four trapezoids with a curved side

(23) $$\begin{array}{c} E_{1}=\left\{\left(x,y\right);x_{1}\leq x\leq x_{2},0\leq y\leq g\left(x\right)\right\},\\ E_{2}=\left\{\left(x,y\right);h\left(y\right)\leq x\leq 1,y_{2}\leq y\leq y_{3}\right\},\\ E_{3}=\left\{\left(x,y\right);x_{4}\leq x\leq x_{3},\;g^{*}\left(x\right)\leq y\leq 1\right\},\\ E_{4}=\left\{\left(x,y\right);0\leq x\leq h^{*}\left(y\right),y_{1}\leq y\leq y_{4}\right\}, \end{array}$$

where *g* ∈ *C* ^*∞*^([*x*_1_, *x*_2_]), *h* ∈ *C* ^*∞*^([*y*_2_, *y*_3_]), g* ∈ *C* ^*∞*^([*x*_4_, *x*_3_]), and h* ∈ *C* ^*∞*^([*y*_1_, *y*_4_]) and

(24) $$\begin{array}{c} 0<g\left(x\right)<1\;\left(x_{1}\leq x\leq x_{2}\right),\\ 0<h\left(y\right)<1\;\left(y_{2}\leq y\leq y_{3}\right),\\ 0<g^{*}\left(x\right)<1\;\left(x_{4}\leq x\leq x_{3}\right),\\ 0<h^{*}\left(y\right)<1\;\left(y_{1}\leq y\leq y_{4}\right). \end{array}$$

**Figure 1:**
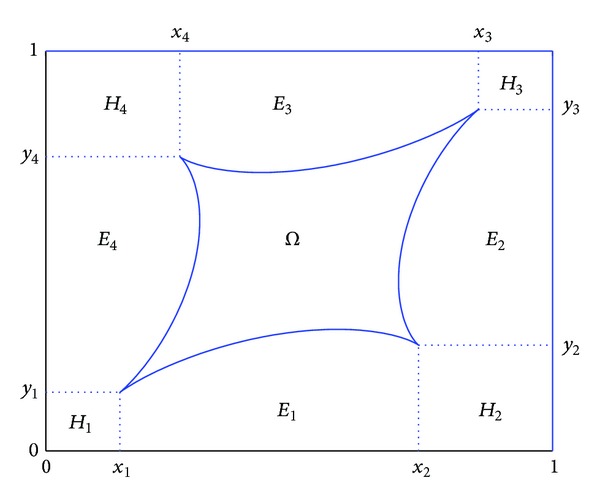
Partition of the complement of the domain *Ω*.

From this, we know that *T* can be expressed into a disjoint union as follows:

(25) $$\begin{array}{c} T=\Omega ⋃\left(\overset{4}{\underset{1}{⋃}}E_{\nu }\right)‍⋃\left(\overset{4}{\underset{1}{⋃}}H_{\nu }\right), \end{array}$$

where each *E*_*ν*_ is a trapezoid with a curved side and each *H*_*ν*_ is a rectangle (see Figure 1).

In Sections 3.2 and 3.3 we will extend *f* to each *E*_*ν*_ and continue to extend to each *H*_*ν*_ such that the obtained extension *F* satisfies the conditions of Theorem 1.

### 3.2. Smooth Extension to Each Trapezoid *E*_*ν*_ with a Curved Side

By (23), the trapezoid *E*_1_ with a curved side *y* = *g*(*x*)(*x*_1_ ≤ *x* ≤ *x*_2_) is represented as

(26) $$\begin{array}{c} E_{1}=\left\{\left(x,y\right):x_{1}\leq x\leq x_{2},0\leq y\leq g\left(x\right)\right\}. \end{array}$$

We define two sequences of functions {*a*_*k*,1_(*x*, *y*)}_0_^*∞*^ and {*b*_*k*,1_(*x*, *y*)}_0_^*∞*^ as follows:

(27) a0,1(x,y)=yg(x),  b0,1(x,y)=y−g(x)−g(x),ak,1(x,y)=(y−g(x))kk!(yg(x))k+1,bk,1(x,y)=ykk!(y−g(x)−g(x))k+1, k=1,2,….

By (27), we deduce that for *x*_1_ ≤ *x* ≤ *x*_2_,

(28) ∂lak,1∂yl(x,g(x))=0      (0≤l≤k−1),∂kak,1∂yk(x,g(x))=1; (∂lak,1)∂yl(x,0)=0 (0≤l≤k),∂lbk,1∂yl(x,g(x))=0      (0≤l≤k);  ∂lbk,1∂yl(x,0)=0      (0≤l≤k−1), (∂kbk,1)∂yk(x,0)=1.

On *E*_1_, we define a sequence of functions {*S*_1_^(*k*)^(*x*, *y*)}_0_^*∞*^ by induction.

Let

(29) S1(0)(x,y)=f(x,g(x))a0,1(x,y)(x1≤x≤x2, 0≤y≤g(x)).

Then, by (27),

(30) S1(0)(x,0)=0,  S1(0)(x,g(x))=f(x,g(x)),(x1≤x≤x2).

Let

(31) S1(1)(x,y)=S1(0)(x,y)+a1,1(x,y)(∂f∂y(x,g(x))−∂S1(0)∂y(x,g(x)))−b1,1(x,y)∂S1(0)∂y(x,0)(x1≤x≤x2,   0≤y≤g(x)).

Then, by (27)–(30), we obtain that, for *x*_1_ ≤ *x* ≤ *x*_2_,

(32) $$\begin{array}{c} S_{1}^{\left(1\right)}\left(x,g\left(x\right)\right)=f\left(x,g\left(x\right)\right),\\ \frac{\partial S_{1}^{\left(1\right)}}{\partial y}\left(x,g\left(x\right)\right)=\frac{\partial f}{\partial y}\left(x,g\left(x\right)\right),\\ S_{1}^{\left(1\right)}\left(x,0\right)=0,\;\;\frac{\partial S_{1}^{\left(1\right)}}{\partial y}\left(x,0\right)=0. \end{array}$$

In general, let

(33) S1(k)(x,y)=S1(k−1)(x,y)+ak,1(x,y)×(∂kf∂yk(x,g(x))−∂kS1(k−1)∂yk(x,g(x)))−bk,1(x,y)∂kS1(k−1)∂yk(x,0)(x1≤x≤x2, 0≤y≤g(x)).

**Lemma 4**

For any *k* ∈ *ℤ*_+_, one has *S*_1_^(*k*)^ ∈ *C* ^*∞*^(*E*_1_) and

(34) $$\begin{array}{c} S_{1}^{\left(k\right)}\left(x,y\right)=\overset{2k+1}{\underset{j=0}{\sum }}\zeta_{j,1}\left(x\right)y^{j}‍,\;\left(x,y\right)\in E_{1}. \end{array}$$

**Proof**

Since *f* ∈ *C* ^*∞*^(*Ω*) and *g* ∈ *C* ^*∞*^([*x*_1_, *x*_2_]), and *g*(*x*) > 0  (*x*_1_ ≤ *x* ≤ *x*_2_), by the above construction, we know that *S*_1_^(*k*)^ ∈ *C* ^*∞*^(*E*_1_) for any *k* = 0,1,….

For *k* = 0, since

(35) $$\begin{array}{c} S_{1}^{\left(0\right)}\left(x,y\right)=f\left(x,g\left(x\right)\right)a_{0,1}\left(x,y\right)=\frac{f\left(x,g\left(x\right)\right)}{g\left(x\right)}y, \end{array}$$

(34) holds. We assume that (34) holds for *k* = *l* − 1; that is,

(36) $$\begin{array}{c} S_{1}^{\left(l-1\right)}\left(x,y\right)=\overset{2l-1}{\underset{j=0}{\sum }}\zeta_{j,1}^{\left(l-1\right)}\left(x\right)y^{j}. \end{array}$$

This implies that

(37) $$\begin{array}{c} \frac{\partial^{l}S_{1}^{\left(l-1\right)}}{\partial y^{l}}\left(x,g\left(x\right)\right)=\overset{2l-1}{\underset{j=l}{\sum }}\frac{j!}{\left(j-l\right)!}\;\zeta_{j,1}^{\left(l-1\right)}\left(x\right){\left(g\left(x\right)\right)}^{j-l},\\ \frac{\partial^{l}S_{1}^{\left(l-1\right)}}{\partial y^{l}}\left(x,0\right)=l!\zeta_{l,1}^{\left(l-1\right)}\left(x\right). \end{array}$$

Again, notice that *a*_*l*,1_(*x*, *y*) and *b*_*l*,1_(*x*, *y*) are polynomials of *y* whose degrees are both 2*l* + 1. From this and (33), it follows that (34) holds for *k* = *l*. By induction, (34) holds for all *k*.  Lemma 4 is proved.

Below we compute derivatives (∂^*l*^ *S*_1_^(*k*)^/∂*y* ^*l*^)(*x*, *y*)  (0 ≤ *l* ≤ *k*) on the curved side Γ_1_ = {(*x*, *g*(*x*)) : *x*_1_ ≤ *x* ≤ *x*_2_} and the bottom side Δ_1_ = {(*x*, 0) : *x*_1_ ≤ *x* ≤ *x*_2_} of *E*_1_.

**Lemma 5**

Let *S*_1_^(*k*)^(*x*, *y*) be stated as above. For any *k* ∈ *ℤ*_+_, one has

(38) $$\begin{array}{c} \frac{\partial^{l}S_{1}^{\left(k\right)}}{\partial y^{l}}\left(x,g\left(x\right)\right)=\frac{\partial^{l}f}{\partial y^{l}}\left(x,g\left(x\right)\right),\\ \frac{\partial^{l}S_{1}^{\left(k\right)}}{\partial y^{l}}\left(x,0\right)=0\;\left(x_{1}\leq x\leq x_{2},\;\;0\leq l\leq k\right). \end{array}$$

**Proof**

By (30), We have known that, for *k* = 0, (38) holds.

Now we assume that (38) holds for *k* − 1.

For *x*_1_ ≤ *x* ≤ *x*_2_, by (33), we have

(39) ∂lS1(k)∂yl(x,  g(x))=∂lS1(k−1)∂yl(x,g(x))+∂lak,1∂yl(x,g(x)) ×(∂kf∂yk(x,g(x))−∂kS1(k−1)∂yk(x,g(x))) −∂lbk,1∂yl(x,g(x))∂kS1(k−1)∂yk(x,0),(0≤l≤k).

For *l* = 0,1,…, *k* − 1, by the assumption of induction, we have

(40) $$\begin{array}{c} \frac{\partial^{l}S_{1}^{\left(k-1\right)}}{\partial y^{l}}\left(x,g\left(x\right)\right)=\frac{\partial^{l}f}{\partial y^{l}}\left(x,g\left(x\right)\right). \end{array}$$

By (28), we have (∂^*l*^ *a*_*k*,1_/∂*y* ^*l*^)(*x*, *g*(*x*)) = 0, (∂^*l*^ *b*_*k*,1_/∂*y* ^*l*^)(*x*, *g*(*x*)) = 0. So we get

(41) $$\begin{array}{c} \frac{\partial^{l}S_{1}^{\left(k\right)}}{\partial y^{l}}\left(x,g\left(x\right)\right)=\frac{\partial^{l}f}{\partial y^{l}}\left(x,g\left(x\right)\right). \end{array}$$

For *l* = *k*, note that (∂^*k*^ *a*_*k*,1_/∂*y* ^*k*^)(*x*, *g*(*x*)) = 1 and (∂^*k*^ *b*_*k*,1_/∂*y* ^*k*^)(*x*, *g*(*x*)) = 0. By (39), we get

(42) ∂kS1(k)∂yk(x,g(x)) =∂kS1(k−1)∂yk(x,g(x))  +(  ∂kf∂yk(x,g(x))−∂kS1(k−1)∂yk(x,g(x))) =∂kf∂yk(x,  g(x)).

The first formula of (38) holds for *k*.

By (33), we have

(43) ∂lS1(k)∂yl(x,0)=∂lS1(k−1)∂yl(x,0)+∂lak,1∂yl(x,0) ×(∂kf∂yk(x,g(x))−∂kS1(k−1)∂yk(x,g(x))) −∂lbk,1∂yl(x,0)∂kS1(k−1)∂yk(x,0),  (x1≤x≤x2,   0≤l≤k).

For *l* = 0,…, *k* − 1, by the assumption of induction and (28), we have (∂^*l*^ *S*_1_^(*k*−1)^/∂*y* ^*l*^)(*x*, 0) = 0 and (∂^*l*^ *a*_*k*,1_/∂*y* ^*l*^)(*x*, 0) = (∂^*l*^ *b*_*k*,1_/∂*y* ^*l*^)(*x*, 0) = 0. So

(44) $$\begin{array}{c} \frac{\partial^{l}S_{1}^{\left(k\right)}}{\partial y^{l}}\left(x,0\right)=0. \end{array}$$

For *l* = *k*, since (∂^*k*^ *a*_*k*,1_/∂*y* ^*k*^)(*x*, 0) = 0, (∂^*k*^ *b*_*k*,1_/∂*y* ^*k*^)(*x*, 0) = 1, by (43), we have

(45) $$\begin{array}{c} \frac{\partial^{k}S_{1}^{\left(k\right)}}{\partial y^{k}}\left(x,0\right)=\frac{\partial^{k}S_{1}^{\left(k-1\right)}}{\partial y^{k}}\left(x,0\right)-\frac{\partial^{k}S_{1}^{\left(k-1\right)}}{\partial y^{k}}\left(x,0\right)=0. \end{array}$$

The second formula of (38) holds. By induction, (38) holds for all *k*. From this, we get Lemma 5.

Now we compute the mixed derivatives of *S*_1_^(*k*)^(*x*, *y*) on the curved side Γ_1_ and bottom side Δ_1_ of *E*_1_.

**Lemma 6**

Let Γ_1_ and Δ_1_ be the curved side and the bottom side of *E*_1_, respectively. Then, for *k* ∈ *ℤ*_+_,

- (∂^*i*+*j*^ *S*_1_^(*k*)^/∂*x* ^*i*^∂*y* ^*j*^)(*x*, *y*) = (∂^*i*+*j*^ *f*/∂*x* ^*i*^∂*y* ^*j*^)(*x*, *y*)  ((*x*, *y*) ∈ Γ_1_),
- (∂^*i*+*j*^ *S*_1_^(*k*)^/∂*x* ^*i*^∂*y* ^*j*^)(*x*, *y*) = 0  ((*x*, *y*) ∈ Δ_1_),

where 0 ≤ *i* + *j* ≤ *k*.

**Proof**

Let *x*_1_ ≤ *x* ≤ *x*_2_. Then we have

(46) ddx(∂l−1f∂yl−1(x,g(x)))=∂lf∂x∂yl−1(x,g(x)) +∂lf∂yl(x,g(x))g′(x), (l≥1).

By the Newton-Leibniz formula, we have

(47) ∂l−1f∂yl−1(x,g(x)) =∂l−1f∂yl−1(x1,g(x1))  +∫x1x(∂lf∂x∂yl−1(t,g(t))+∂lf∂yl(t,g(t))g′(t))dt.

Similarly, replacing *f* by *S*_1_^(*k*)^ in this formula, we have

(48) ∂l−1S1(k)∂yl−1(x,g(x)) =∂l−1S1(k)∂yl−1(x1,g(x1))  +∫x1x(∂lS1(k)∂x∂yl−1(t,g(t))+∂lS1(k)∂yl(t,g(t))g′(t))dt,(l≥1).

From this and Lemma 5, it follows that, for any *x*_1_ ≤ *x* ≤ *x*_2_, we have

(49) ∫x1x∂lS1(k)∂x∂yl−1(t,g(t))dt‍ =∫x1x∂lf∂x∂yl−1(t,g(t))dt ‍(1≤l≤k).

Finding derivatives on the both sides of this formula, we get

(50) $$\begin{array}{c} \frac{\partial^{l}S_{1}^{\left(k\right)}}{\partial x\partial y^{l-1}}\left(x,g\left(x\right)\right)=\frac{\partial^{l}f}{\partial x\partial y^{l-1}}\left(x,g\left(x\right)\right)\;\left(1\leq l\leq k\right). \end{array}$$

Now we start from the equality

(51) ddx(∂l−1f∂x∂yl−2(x,g(x)))=∂lf∂x2∂yl−2(x,g(x)) +∂lf∂x∂yl−1(x,g(x))g′(x),(l≥2).

Similar to the argument from (46) to (50), we get

(52) $$\begin{array}{c} \frac{\partial^{l}S_{1}^{\left(k\right)}}{\partial x^{2}\partial y^{l-2}}\left(x,g\left(x\right)\right)=\frac{\partial^{l}f}{\partial x^{2}\partial y^{l-2}}\left(x,g\left(x\right)\right)\;\left(2\leq l\leq k\right). \end{array}$$

Continuing this procedure, we deduce that (i) holds for 0 < *i* + *j* ≤ *k*. Letting *l* = 0 in Lemma 5, we have *S*_1_^(*k*)^(*x*, *g*(*x*)) = *f*(*x*, *g*(*x*)); that is, (i) holds for *i* = *j* = 0. So we get (i).

By Lemma 5, (∂^*j*^ *S*_1_^(*k*)^/∂*y* ^*j*^)(*x*, 0) = 0  (0 ≤ *j* ≤ *k*). From this and *S*_1_^(*k*)^ ∈ *C* ^*∞*^(*E*_1_), we have

(53) $$\begin{array}{c} \frac{\partial^{i+j}S_{1}^{\left(k\right)}}{\partial x^{i}\partial y^{j}}\left(x,0\right)=0\;\left(0\leq i+j\leq k\right), \end{array}$$

so (ii) holds.  Lemma 6 is proved.

From this, we get the following.

**Lemma 7**

For any positive integer *r*, denote *l*_*r*_ = *r*(*r* + 1)(*r* + 2)(*r* + 3). Let

(54) $$\begin{array}{c} F\left(x,y\right)=\{\begin{array}{cc} S_{1}^{\left(l_{r}\right)}\left(x,y\right), & \left(x,y\right)\in E_{1},\\ f\left(x,y\right), & \left(x,y\right)\in \Omega . \end{array} \end{array}$$

Then (i) *F* ∈ *C* ^*l*_*r*_^(*Ω*⋃*E*_1_)   and   *F*(*x*, *y*) = *f*(*x*, *y*) ((*x*, *y*) ∈ *Ω*); (ii) (∂^*i*+*j*^ *F*/∂*x* ^*i*^∂*y* ^*j*^)(*x*, *y*) = 0((*x*, *y*)∈(*E*_1_⋂∂*T*), 0 ≤ *i* + *j* ≤ *l*_*r*_).

**Proof**

By the assumption *f* ∈ *C* ^*∞*^(*Ω*), Lemma 4: *S*_1_^(*k*)^ ∈ *C* ^*∞*^(*E*_1_), and Lemma 6(i):

(55) ∂i+jS1(k)∂xi∂yj(x,y)=∂i+jf∂xi∂yj(x,y)((x,y)∈Γ1, 0≤i+j≤k),

where Γ_1_ = *Ω*⋂*E*_1_, we get (i). By Lemma 6(ii) and *E*_1_⋂∂*T* = Δ_1_, we get (ii).  Lemma 7 is proved.

For *ν* = 2,3, 4, by using a similar method, we define *S*_*ν*_^(*k*)^(*x*, *y*) on the each trapezoid *E*_*ν*_ with a curve side. The representations of *S*_*ν*_^(*k*)^(*x*, *y*) are stated in Section 4.1.

**Lemma 8**

For any *ν* = 1,2, 3,4, let

(56) $$\begin{array}{c} F\left(x,y\right)=\{\begin{array}{cc} S_{\nu }^{\left(l_{r}\right)}\left(x,y\right), & \left(x,y\right)\in E_{\nu },\\ f\left(x,y\right), & \left(x,y\right)\in \Omega , \end{array} \end{array}$$

where *l*_*r*_ = *r*(*r* + 1)(*r* + 2)(*r* + 3). Then, for *ν* = 1,2, 3,4, one has the following:

(i) *F* ∈ *C* ^*l*_*r*_^(*Ω*⋃*E*_*ν*_  );
(ii) (∂^*i*+*j*^ *F*/∂*x* ^*i*^∂*y* ^*j*^)(*x*, *y*) = 0, (*x*, *y*)∈(*E*_*ν*_⋂∂*T*) for 0 ≤ *i* + *j* ≤ *l*_*r*_;
(iii) *F*(*x*, *y*) can be expressed in the form:
  (57) $$\begin{array}{c} F\left(x,y\right)=\overset{2l_{r}+1}{\underset{j=0}{\sum }}\zeta_{j,1}\left(x\right)y^{j},\;\left(x,y\right)\in E_{1},\\ F\left(x,y\right)=\overset{2l_{r}+1}{\underset{j=0}{\sum }}\zeta_{j,2}\left(y\right)x^{j},\;\left(x,y\right)\in E_{2},\\ F\left(x,y\right)=\overset{2l_{r}+1}{\underset{j=0}{\sum }}\zeta_{j,3}\left(x\right)y^{j},\;\left(x,y\right)\in E_{3},\\ F\left(x,y\right)=\overset{2l_{r}+1}{\underset{j=0}{\sum }}\zeta_{j,4}\left(y\right)x^{j},\;\left(x,y\right)\in E_{4}. \end{array}$$

**Proof**

By Lemma 7, we have

(58) F∈Clr(Ω⋃E1),  ∂i+jF∂xi∂yj(x,y)=0,((x,y)∈(E1⋂∂T),0≤i+j≤lr).

Similar to the argument of Lemma 7, for *ν* = 2,3, 4, we have

(59) F∈Clr(Ω⋃Eν),  ∂i+jF∂xi∂yj(x,y)=0,((x,y)∈(Eν⋂∂T),  0≤i+j≤lr).

From this, we get (i) and (ii).

The proof of (iii) is similar to the argument of Lemma 4(iii). Lemma 8 is proved.

### 3.3. Smooth Extension to Each Rectangle *H*_*ν*_

We have completed the smooth extension of *f* to each trapezoid *E*_*ν*_ with a curved side. In this subsection we complete the smooth extension of the obtained function *F* to each rectangle *H*_*ν*_. First we consider the smooth extension of *F* to *H*_1_. We divide this procedure in two steps.

**Step 1**

In Lemma 8, we know that *F*(*x*, *y*) = *S*_4_^*l*_*r*_^(*x*, *y*) on *E*_4_. Now we construct the smooth extension of *S*_4_^(*l*_*r*_)^(*x*, *y*) from *E*_4_ to *H*_1_, where *S*_4_^(*l*_*r*_)^(*x*, *y*) is stated in Section 4.2 and *l*_*r*_ = *r*(*r* + 1)(*r* + 2)(*r* + 3).

Let

(60) αk,11(y)=(y−y1)kk!(yy1)k+1,βk,11(y)=ykk!(y−y1−y1)k+1, k=0,1,…,

and let

(61) M1(0)(x,y)=S4(lr)(x,y1)α0,11(y),M1(k)(x,y)=M1(k−1)(x,y)+αk,11(y)×(∂kS4(lr)∂yk(x,y1)−∂kM1(k−1)∂yk(x,y1))−βk,11(y)∂kM1(k−1)∂yk(x,0),k=1,2,…,τr  ((x,y)∈H1),

where *τ*_*r*_ = *r*(*r* + 2).

**Lemma 9**

Let {*J*_1,*l*_}_1_^4^ be four sides of the rectangle *H*_1_:

(62) $$\begin{array}{c} J_{1,1}=\left\{\left(x,y_{1}\right),0\leq x\leq x_{1}\right\},\\ J_{1,2}=\left\{\left(0,y\right),0\leq y\leq y_{1}\right\},\\ J_{1,3}=\left\{\left(x,0\right),0\leq x\leq x_{1}\right\},\\ J_{1,4}=\left\{\left(x_{1},y\right),0\leq y\leq y_{1}\right\}. \end{array}$$

Then one has the following

- *M*_1_^(*τ*_*r*_)^(*x*, *y*) = ∑_*i*,*j*=0_^2*l*_*r*_+1^ *d*_*i*,*j*_^(1)^ *x* ^*i*^ *y* ^*j*^, where *d*_*i*,*j*_^(1)^ is a constant;
- (∂^*i*+*j*^ *M*_1_^(*τ*_*r*_)^/∂*x* ^*i*^∂*y* ^*j*^)(*x*, *y*) = (∂^*i*+*j*^ *S*_4_^(*l*_*r*_)^/∂*x* ^*i*^∂*y* ^*j*^)(*x*, *y*)  ((*x*, *y*) ∈ *J*_1,1_);
- (∂^*i*+*j*^ *M*_1_^(*τ*_*r*_)^/∂*x* ^*i*^∂*y* ^*j*^)(*x*, *y*) = 0  ((*x*, *y*) ∈ *J*_1,2_);
- (∂^*i*+*j*^ *M*_1_^(*τ*_*r*_)^/∂*x* ^*i*^∂*y* ^*j*^)(*x*, *y*) = 0  ((*x*, *y*) ∈ *J*_1,3_),

where 0 ≤ *i* + *j* ≤ *τ*_*r*_.

**Proof**

By Lemma 8(iii), we have

(63) $$\begin{array}{c} S_{4}^{\left(l_{r}\right)}\left(x,y\right)=\overset{2l_{r}+1}{\underset{j=0}{\sum }}\zeta_{j,4}\left(y\right)x^{j}‍. \end{array}$$

So (∂^*k*^ *S*_4_^(*l*_*r*_)^/∂*y* ^*k*^)(*x*, *y*_1_) is a polynomial of degree 2*l*_*r*_ + 1 with respect to *x*. Since *α*_*τ*_*r*_,11_(*y*) and *β*_*τ*_*r*_,11_(*y*) are both polynomials of degree 2*τ*_*r*_ + 1, (i) follows from (61).

Similar to the argument of Lemma 6, we get (ii) and (iv).

Since (0, *y*_1_)∈(*E*_4_⋂∂*T*), by Lemma 7, we have

(64) $$\begin{array}{c} \frac{\partial^{i+j}S_{4}^{\left(l_{r}\right)}}{\partial x^{i}\partial y^{j}}\left(0,y_{1}\right)=\frac{\partial^{i+j}F}{\partial x^{i}\partial y^{j}}\left(0,y_{1}\right)=0,\;\left(0\leq i+j\leq l_{r}\right). \end{array}$$

By the definition of *M*_1_^(0)^ and (64), we have

(65) ∂i+jM1(0)∂xi∂yj(0,y)=∂iS4(lr)∂xi(0,y1)djα0,11dyj(y)=0,(0≤i+j≤lr,y∈ℝ).

We assume that

(66) $$\begin{array}{c} \frac{\partial^{i+j}M_{1}^{\left(k-1\right)}}{\partial x^{i}\partial y^{j}}\left(0,y\right)=0,\;\left(0\leq i+j\leq l_{r}-\frac{1}{2}k\left(k-1\right)\right). \end{array}$$

By (61), we get

(67) ∂i+jM1(k)∂xi∂yj(0,y)=∂i+jM1(k−1)∂xi∂yj(0,y)+djαk,11dyj(y) ×(∂k+iS4(lr)∂xi∂yk(0,y1)−∂k+iM1(k−1)∂xi∂yk(0,y1)) −djβk,11dyj(y)∂k+iM1(k−1)∂xi∂yk(0,0).

for 0 ≤ *i* + *j* ≤ *l*_*r*_ − (1/2)*k*(*k* + 1), we have 0 ≤ *i* + *j* ≤ *l*_*r*_ − (1/2)*k*(*k* − 1) and 0 ≤ *i* + *k* ≤ *l*_*r*_ − (1/2)*k*(*k* − 1). Again, by the assumption of induction, we get

(68) $$\begin{array}{c} \frac{\partial^{i+j}M_{1}^{\left(k-1\right)}}{\partial x^{i}\partial y^{j}}\left(0,y\right)=0,\;\;\frac{\partial^{k+i}M_{1}^{\left(k-1\right)}}{\partial x^{i}\partial y^{k}}\left(0,y_{1}\right)=0. \end{array}$$

By (64), we have (∂^*k*+*i*^ *S*_4_^(*l*_*r*_)^/∂*x* ^*i*^∂*y* ^*k*^)(0, *y*_1_) = 0. From this and (67), we get

(69) ∂i+jM1(k)∂xi∂yj(0,y)=0,(0≤y≤y1,   0≤i+j≤lr−k2(k+1)).

Taking *k* = *τ*_*r*_, we have

(70) ∂i+jM1(τr)∂xi∂yj(0,y)=0,(0≤y≤y1,   0≤i+j≤lr−τr2(τr+1)).

Since *l*_*r*_ − (*τ*_*r*_/2)(*τ*_*r*_ + 1) = (*l*_*r*_/2) ≥ *τ*_*r*_, we get (iii).  Lemma 9 is proved.

**Step 2**

In Lemma 8, we know that *F*(*x*, *y*) = *S*_1_^*l*_*r*_^(*x*, *y*) on *E*_1_. We consider the difference *S*_1_^(*l*_*r*_)^(*x*, *y*) − *M*_1_^(*τ*_*r*_)^(*x*, *y*). Obviously, it is infinitely many time differentiable on *E*_1_ since *M*_1_^(*τ*_*r*_)^(*x*, *y*) is a polynomial. Now we construct its smooth extension from *E*_1_ to the rectangle *H*_1_ as follows. Let

(71) αk,14(x)=(x−x1)kk!(xx1)k+1,  βk,14(x)=xkk!(x−x1−x1)k+1, k=0,1,…,

and let

(72) N1(0)(x,y)=(S1(lr)(x1,y)−M1(τr)(x1,y))α0,14(x),N1(k)(x,y)=N1(k−1)(x,y)+αk,14(x)×(∂k(S1(lr)−M1(τr))∂xk(x1,y)−∂kN1(k−1)∂xk(x1,y))−βk,14(x)∂kN1(k−1)∂xk(0,y),k=0,1,…,r,  ((x,y)∈H1).

From this, we obtain the following.

**Lemma 10**

*N*_1_^(*r*)^(*x*, *y*) possesses the following properties:

- (∂^*i*+*j*^ *N*_1_^(*r*)^/∂*x* ^*i*^∂*y* ^*j*^)(*x*, *y*) = (∂^*i*+*j*^ *S*_1_^(*l*_*r*_)^/∂*x* ^*i*^∂*y* ^*j*^)(*x*, *y*) − (∂^*i*+*j*^ *M*_1_^(*τ*_*r*_)^/∂*x* ^*i*^∂*y* ^*j*^)(*x*, *y*) on *J*_1,4_;
- (∂^*i*+*j*^ *N*_1_^(*r*)^/∂*x* ^*i*^∂*y* ^*j*^)(*x*, *y*) = 0 on *J*_1,2_;
- (∂^*i*+*j*^ *N*_1_^(*r*)^/∂*x* ^*i*^∂*y* ^*j*^)(*x*, *y*) = 0 on *J*_1,1_;
- (∂^*i*+*j*^ *N*_1_^(*r*)^/∂*x* ^*i*^∂*y* ^*j*^)(*x*, *y*) = 0 on *J*_1,3_, where 0 ≤ *i* + *j* ≤ *r* and {*J*_1,*ν*_}_1_^4^ are stated in (62);
- *N*_1_^(*r*)^(*x*, *y*) = ∑_*i*,*j*=0_^2*l*_*r*_+1^ *τ*_*i*,*j*_^(1)^ *x* ^*i*^ *y* ^*j*^, where *τ*_*i*,*j*_^(1)^ is a constant.

**Proof**

The arguments similar to Lemma 9(ii) and (iv) give the conclusions (i) and (ii) of this theorem. Now we prove (iii) and (iv).

By Lemma 6(i) and Lemma 9(ii), as well as *l*_*r*_ ≥ *τ*_*r*_, we get that, for 0 ≤ *i* + *j* ≤ *τ*_*r*_,

(73) ∂i+jS1(lr)∂xi∂yj(x1,y1)=∂i+jf∂xi∂yj(x1,y1)=∂i+jS4(lr)∂xi∂yj(x1,y1)=∂i+jM1(τr)∂xi∂yj(x1,y1).

So we have

(74) ∂i+jN1(0)∂xi∂yj(x,y1)=∂j(S1(lr)−M1(τr))∂yj(x1,y1)diα0,14dxi(x)=0,(0≤x≤x1,  0≤i+j≤τr).

Now we assume that

(75) ∂i+jN1(k−1)∂xi∂yj(x,y1)=0,(0≤x≤x1,  0≤i+j≤τr−k2(k−1)).

By (72) and (73),

(76) ∂i+jN1(k)∂xi∂yj(x,y1)=∂i+jN1(k−1)∂xi∂yj(x,y1)+diαk,14dxi(x)×(∂k+j(S1(lr)−M1(τr))∂xk∂yj(x1,y1)−∂k+jN1(k−1)∂xk∂yj(x1,y1))−diβk,14dxi(x)∂k+jN1(k−1)∂xk∂yj(0,y1)=0,(0≤x≤x1,  0≤i+j≤τr−k(k+1)2).

By induction, we get

(77) ∂i+jN1(k)∂xi∂yj(x,y1)=0,(0≤x≤x1,  0≤i+j≤τr−k(k+1)2).

From this and *τ*_*r*_ − (1/2)*r*(*r* + 1) ≥ *r*, we get (iii). By Lemma 6(ii) and Lemma 9(iii), we get that

(78) $$\begin{array}{c} \frac{\partial^{i+j}S_{1}^{\left(l_{r}\right)}}{\partial x^{i}\partial y^{j}}\left(x,0\right)=0,\;\left(0\leq i+j\leq l_{r}\right),\;\;\;\\ \frac{\partial^{i+j}M_{1}^{\left(\tau_{r}\right)}}{\partial x^{i}\partial y^{j}}\left(x,0\right)=0,\;\left(0\leq i+j\leq \tau_{r}\right). \end{array}$$

From this and (72), by using an argument similar to the proof of (iii), we get (iv).

By Lemma 8(iii) and Lemma 9(i), we deduce that (*S*_1_^(*l*_*r*_)^ − *M*_1_^(*τ*_*r*_)^)(*x*_1_, *y*) is a polynomial of degree 2*l*_*r*_ + 1 with respect to *y*. From this and (72), we get (v).  Lemma 10 is proved.

By Lemmas 9 and 10, we obtain that for 0 ≤ *i* + *j* ≤ *r*,

(79) $$\begin{array}{c} \;\;\;\;\frac{\partial^{i+j}\left(M_{1}^{\left(\tau_{r}\right)}+N_{1}^{\left(r\right)}\right)}{\partial x^{i}\partial y^{j}}\left(x,y\right)=\frac{\partial^{i+j}S_{4}^{\left(l_{r}\right)}}{\partial x^{i}\partial y^{j}}\left(x,y\right)\;\mathrm{on}\;J_{1,1}, \end{array}$$

(80) $$\begin{array}{c} \frac{\partial^{i+j}\left(M_{1}^{\left(\tau_{r}\right)}+N_{1}^{\left(r\right)}\right)}{\partial x^{i}\partial y^{j}}\left(x,y\right)=0\;\mathrm{on}\;J_{1,2}⋃J_{1,3}, \end{array}$$

(81) $$\begin{array}{c} \;\;\;\frac{\partial^{i+j}\left(M_{1}^{\left(\tau_{r}\right)}+N_{1}^{\left(r\right)}\right)}{\partial x^{i}\partial y^{j}}\left(x,y\right)=\frac{\partial^{i+j}S_{1}^{\left(l_{r}\right)}}{\partial x^{i}\partial y^{j}}\left(x,y\right)\;\mathrm{on}\;J_{1,4}. \end{array}$$

**Lemma 11**

Let

(82) $$\begin{array}{c} F\left(x,y\right)=\{\begin{array}{cc} f\left(x,y\right), & \left(x,y\right)\in \Omega ,\\ S_{1}^{\left(l_{r}\right)}\left(x,y\right), & \left(x,y\right)\in E_{1},\\ S_{4}^{\left(l_{r}\right)}\left(x,y\right), & \left(x,y\right)\in E_{4},\\ M_{1}^{\left(\tau_{r}\right)}\left(x,y\right)+N_{1}^{\left(r\right)}\left(x,y\right), & \left(x,y\right)\in H_{1}. \end{array} \end{array}$$

Then one has

- *F* ∈ *C* ^*r*^(*Ω*⋃*E*_1_⋃*E*_4_⋃*H*_1_),
- (∂^*i*+*j*^ *F*/∂*x* ^*i*^∂*y* ^*j*^)(*x*, *y*) = 0,   (*x*, *y*) ∈ ((*E*_1_⋃*E*_4_⋃*H*_1_)⋂∂*T*) for 0 ≤ *i* + *j* ≤ *r*;
- *F*(*x*, *y*) = ∑_*i*,*j*=0_^2*l*_*r*_+1^ *c*_*ij*_^(1)^ *x* ^*i*^ *y* ^*j*^((*x*, *y*) ∈ *H*_1_), where each *c*_*ij*_^(1)^ is constant.

**Proof**

By Lemma 7, we have *F* ∈ *C* ^*r*^(*Ω*⋃*E*_1_⋃*E*_4_). Since *S*_1_^(*l*_*r*_)^ ∈ *C* ^*r*^(*E*_1_),

(83) $$\begin{array}{c} M_{1}^{\left(\tau_{r}\right)}+N_{1}^{\left(r\right)}\in C^{r}\left(H_{1}\right),\;E_{1}⋂H_{1}=J_{1,4}, \end{array}$$

by (81), we deduce that *F* ∈ *C* ^*r*^(*E*_1_⋃*H*_1_). Since *S*_4_^(*l*_*r*_)^ ∈ *C* ^*r*^(*E*_4_),

(84) $$\begin{array}{c} M_{1}^{\left(\tau_{r}\right)}+N_{1}^{\left(r\right)}\in C^{r}\left(H_{1}\right),\;E_{4}⋂H_{1}=J_{1,1}, \end{array}$$

by (79), we deduce that *F* ∈ *C* ^*r*^(*H*_1_⋃*E*_4_). So we get (i).

By Lemma 8(ii),

(85) $$\begin{array}{c} \frac{\partial^{i+j}F}{\partial x^{i}\partial y^{j}}\left(x,y\right)=0,\;\left(x,y\right)\in \left(\left(E_{1}⋃E_{4}\right)⋂\partial T\right). \end{array}$$

Since *H*_1_⋂∂*T* = *J*_1,2_⋃*J*_1,3_, by (80), we deduce that

(86) $$\begin{array}{c} \frac{\partial^{i+j}F}{\partial x^{i}\partial y^{j}}\left(x,y\right)=0,\;\left(x,y\right)\in \left(H_{1}⋂\partial T\right). \end{array}$$

So we get (ii).

From Lemma 9(i), Lemma 10(v), and *F*(*x*, *y*) = *M*_1_^(*τ*_*r*_)^(*x*, *y*) + *N*_1_^(*r*)^(*x*, *y*) ((*x*, *y*) ∈ *H*_1_), we get (iii). Lemma 11 is proved.

For *ν* = 2,3, 4, by using a similar method, we define *F*(*x*, *y*) = *M*_*ν*_^(*τ*_*r*_)^(*x*, *y*) + *N*_*ν*_^(*r*)^(*x*, *y*) ((*x*, *y*) ∈ *H*_*ν*_), where representations of *M*_*ν*_^(*τ*_*r*_)^(*x*, *y*) and *N*_*ν*_^(*r*)^(*x*, *y*) see Section 4.2.

### 3.4. The Proofs of the Theorems

**Proof of Theorem 1**

Let

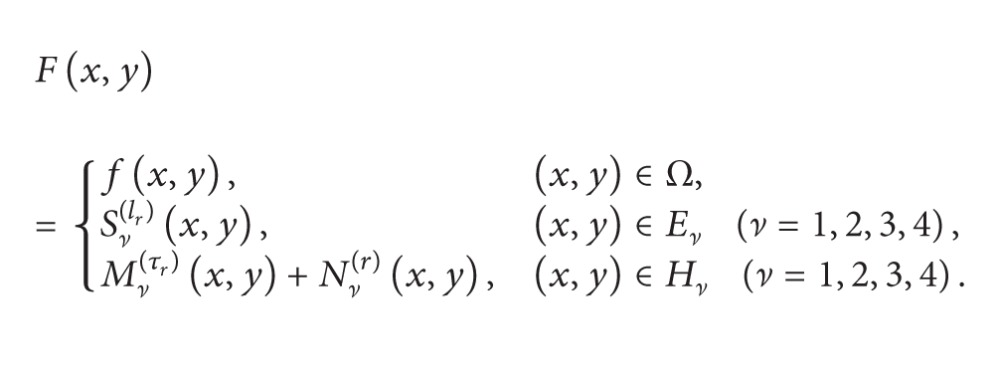
(87)
By (25), *F* has been defined on the unit square *T*. The argument similar to Lemma 11(i)-(ii) shows that

(88) $$\begin{array}{c} F\in C^{r}\left(\Omega ⋃E_{1}⋃E_{4}⋃H_{1}\right);\\ F\in C^{r}\left(\Omega ⋃E_{1}⋃E_{2}⋃H_{2}\right);\\ F\in C^{r}\left(\Omega ⋃E_{2}⋃E_{3}⋃H_{3}\right);\\ F\in C^{r}\left(\Omega ⋃E_{3}⋃E_{4}⋃H_{4}\right); \end{array}$$

and for 0 ≤ *i* + *j* ≤ *r*,

(89) $$\begin{array}{c} \frac{\partial^{i+j}F}{\partial x^{i}\partial y^{j}}\left(x,y\right)=0,\;\left(x,y\right)\in \left(\left(E_{1}⋃E_{4}⋃H_{1}\right)⋂\partial T\right);\\ \frac{\partial^{i+j}F}{\partial x^{i}\partial y^{j}}\left(x,y\right)=0,\;\left(x,y\right)\in \left(\left(E_{1}⋃E_{2}⋃H_{2}\right)⋂\partial T\right);\\ \frac{\partial^{i+j}F}{\partial x^{i}\partial y^{j}}\left(x,y\right)=0,\;\left(x,y\right)\in \left(\left(E_{2}⋃E_{3}⋃H_{3}\right)⋂\partial T\right);\\ \frac{\partial^{i+j}F}{\partial x^{i}\partial y^{j}}\left(x,y\right)=0,\;\left(x,y\right)\in \left(\left(E_{3}⋃E_{4}⋃H_{4}\right)⋂\partial T\right). \end{array}$$

From this and *Ω*⋂∂*T* = *∅*, by (25), we have *F* ∈ *C* ^*r*^(*T*) and (∂^*i*+*j*^ *F*/∂*x* ^*i*^∂*y* ^*j*^)(*x*, *y*) = 0   ((*x*, *y*)∈∂*T*, 0 ≤ *i* + *j* ≤ *r*). So we get (i) and (ii).

Similar to the argument of Lemma 11(iii), we get

(90) $$\begin{array}{c} F\left(x,y\right)=\overset{2l_{r}+1}{\underset{i,j=0}{\sum }}c_{ij}^{\left(\nu \right)}x^{i}y^{j}‍,\;\left(x,y\right)\in H_{\nu }\;\;\left(\nu =1,2,3,4\right), \end{array}$$

where each *c*_*ij*_^(*ν*)^ is a constant. From this and Lemma 8(iii), we know that, on *T*∖*Ω*,   *F*(*x*, *y*) can be expressed locally in the form

(91) $$\begin{array}{c} \overset{2l_{r}+1}{\underset{j=0}{\sum }}\xi_{j}\left(x\right)y^{j}\;\;\text{or}\;\;\overset{2l_{r}+1}{\underset{j=0}{\sum }}\;\eta_{j}\left(x\right)x^{j}\;\;\text{or}\;\overset{2l_{r}+1}{\underset{i,\;j=0}{\sum }}c_{ij}x^{i}y^{j}; \end{array}$$

(iii) holds. We have completed the proof of Theorem 1.

The representation of *F* satisfying the conditions of Theorem 1 is given in Section 4.

**Proof of Theorem 2**

Let *F* be the smooth extension of *f* from *Ω* to *T* which is stated as in Theorem 1. Define *F*_*p*_ by

(92) $$\begin{array}{c} F_{p}\left(x+k,y+l\right)=F\left(x,y\right)\;\left(\left(x,y\right)\in T;\;k,l\in \mathbb{Z}\right). \end{array}$$

Then *F*_*p*_ is a 1-periodic function of ℝ^2^. By Theorem 1, we know that *F*_*p*_ ∈ *C* ^*r*^(*T*) and

(93) $$\begin{array}{c} \frac{\partial^{i+j}F_{p}}{\partial x^{i}\partial y^{j}}\left(x,y\right)=0\;\left(\left(x,y\right)\in \partial T;0\leq i+j\leq r\right). \end{array}$$

Let *T*_*n*_1_,*n*_2__ = [*n*_1_, *n*_1_ + 1]×[*n*_2_, *n*_2_ + 1](*n*_1_, *n*_2_ ∈ *ℤ*). Since *F*_*p*_ is 1-periodic function, we have *F*_*p*_ ∈ *C* ^*r*^(*T*_*n*_1_,*n*_2__) and for any *n*_1_, *n*_2_ ∈ *ℤ*,

(94) $$\begin{array}{c} \frac{\partial^{i+j}F_{p}}{\partial x^{i}\partial y^{j}}\left(x,y\right)=0\;\left(\left(x,y\right)\in \partial T_{n_{1},n_{2}};\;\;0\leq i+j\leq r\right). \end{array}$$

Noticing that ℝ^2^ = ⋃_*n*_1_,*n*_2_∈*ℤ*_ *T*_*n*_1_,*n*_2__, we have *F*_*p*_ ∈ *C* ^*r*^(ℝ^2^). By (92) and Theorem 1(i), we get

(95) $$\begin{array}{c} F_{p}\left(x,y\right)=F\left(x,y\right)=f\left(x,y\right)\;\left(\left(x,y\right)\in \Omega \right). \end{array}$$

Theorem 2 is proved.

**Proof of Theorem 3**

Let *F* be the smooth extension of *f* from *Ω* to *T* which is stated as in Theorem 1. Define *F* ^*c*^ by

(96) $$\begin{array}{c} F^{c}\left(x,y\right)=\{\begin{array}{cc} F\left(x,y\right), & \left(x,y\right)\in T,\\ 0, & \left(x,y\right)\in {\mathbb{R}}^{2}\setminus T. \end{array} \end{array}$$

From Theorem 1(ii), we have

(97) $$\begin{array}{c} \frac{\partial^{i+j}F^{c}}{\partial x^{i}\partial y^{j}}\left(x,y\right)=0\;\left(\left(x,y\right)\in \partial T;0\leq i+j\leq r\right). \end{array}$$

From this and (96), we get *F* ^*c*^(*x*, *y*) ∈ *C* ^*r*^(ℝ^2^). By (96) and Theorem 1(i), we get

(98) $$\begin{array}{c} F^{c}\left(x,y\right)=F\left(x,y\right)=f\left(x,y\right)\;\left(\left(x,y\right)\in \Omega \right). \end{array}$$

Theorem 3 is proved.

## 4. Representation of the Extension *F* Satisfying Theorem 1

Let *f* and *Ω* be stated as in Theorem 1 and let *Ω* be divided as in Section 3.1. The representation of *F* satisfying conditions of Theorem 1 is as follows:

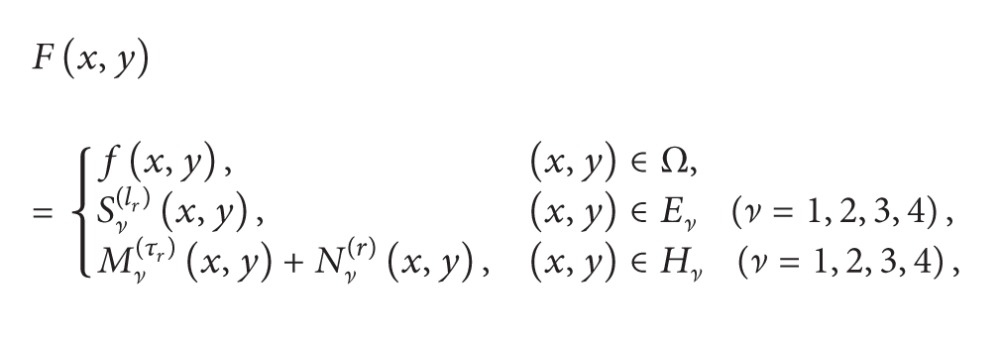
(99)
where

(100) $$\begin{array}{c} T=\Omega ⋃\left(\overset{4}{\underset{1}{⋃}}E_{\nu }\right)⋃\left(\overset{4}{\underset{1}{⋃}}H_{\nu }\right) \end{array}$$

and the rectangles {*H*_*ν*_}_1_^4^ and the trapezoids {*E*_*ν*_}_1_^4^ with a curved side are stated in (22) and (23) and *l*_*r*_ = *r*(*r* + 1)(*r* + 2)(*r* + 3) and *τ*_*r*_ = *r*(*r* + 2).

Below we write out the representations of {*S*_*ν*_^(*k*)^(*x*, *y*)}_1_^4^, {*M*_*ν*_^(*k*)^(*x*, *y*)}_1_^4^, and {*N*_*ν*_^(*k*)^(*x*, *y*)}_1_^4^.

### 4.1. The Construction of Each *S*_*ν*_^(*k*)^(*x*, *y*)

(i) Denote

(101) ak,1(x,y)=(y−g(x))kk!(yg(x))k+1,bk,1(x,y)=ykk!(y−g(x)−g(x))k+1, k=0,1,….

Define *S*_1_^(*k*)^(*x*, *y*) by induction as follows:

(102) S1(0)(x,y)=f(x,g(x))a0,1(x,y),S1(k)(x,y)=S1(k−1)(x,y)+ak,1(x,y)×(∂kf∂yk(x,g(x))−∂kS1(k−1)∂yk(x,g(x)))−bk,1(x,y)∂kS1(k−1)∂yk(x,0),k=1,2,…, ((x,y)∈E1).

(ii) Denote

(103) ak,2(x,y)=(x−h(y))kk!(1−x1−h(y))k+1,bk,2(x,y)=(x−1)kk!(h(y)−xh(y)−1)k+1, k=0,1,….

Define *S*_2_^(*k*)^(*x*, *y*) by induction as follows:

(104) S2(0)(x,y)=f(h(y),y)a0,2(x,y),S2(k)(x,y)=S2(k−1)(x,y)+ak,2(x,y)×(∂kf∂xk(h(y),y)−∂kS2(k−1)∂xk(h(y),y))        −bk,2(x,y)∂kS2(k−1)∂xk(1,y),k=1,2,…, ((x,y)∈E2).

(iii) Denote

(105) ak,3(x,y)=(y−g∗(x))kk!(1−y1−g∗(x))k+1,  bk,3(x,y)=(y−1)kk!(g∗(x)−yg∗(x)−1)k+1, k=0,1,….

Define *S*_3_^(*k*)^(*x*, *y*) by induction as follows:

(106) S3(0)(x,y)=f(x,g∗(x))a0,3(x,y),S3(k)(x,y)=S3(k−1)(x,y)+ak,3(x,y)×(∂kf∂yk(x,g∗(x))−∂kS3(k−1)∂yk(x,g∗(x)))−bk,3(x,y)∂kS3(k−1)∂yk(x,1),k=1,2,… ((x,y)∈E3).

(iv) Denote

(107) ak,4(x,y)=(x−h∗(y))kk!(xh∗(y))k+1,  bk,4(x,y)=xkk!(x−h∗(y)−h∗(y))k+1, k=0,1,….

Define *S*_4_^(*k*)^(*x*, *y*) by induction as follows:

(108) S4(0)(x,y)=f(h∗(y),y)a0,4(x,y),S4(k)(x,y)=S4(k−1)(x,y)+ak,4(x,y)×(∂kf∂xk(h∗(y),y)−∂kS4(k−1)∂xk(h∗(y),y))−bk,4(x,y)∂kS4(k−1)∂xk(0,y),k=1,2,… ((x,y)∈E4).

### 4.2. The Constructions of Each *M*_*ν*_^(*k*)^(*x*, *y*) and *N*_*ν*_^(*k*)^(*x*, *y*)

(i) Denote

(109) αk,11(y)=(y−y1)kk!(yy1)k+1,βk,11(y)=ykk!(y−y1−y1)k+1, k=0,1,….

Define *M*_1_^(*k*)^(*x*, *y*) by induction as follows:

(110) M1(0)(x,y)=S4(lr)(x,y1)α0,11(y),M1(k)(x,y)=M1(k−1)(x,y)+αk,11(y)×(∂kS4(lr)∂yk(x,y1)−∂kM1(k−1)∂yk(x,y1))−βk,11(y)∂kM1(k−1)∂yk(x,0),k=1,2,…,  ((x,y)∈H1).

Denote

(111) αk,14(x)=(x−x1)kk!(xx1)k+1,                                              βk,14(x)=xkk!(x−x1−x1)k+1, k=0,1,….

Define *N*_1_^(*k*)^(*x*, *y*) by induction as follows:

(112) N1(0)(x,y)=(S1(lr)(x1,y)−M1(2r)(x1,y))α0,14(x)N1(k)(x,y)=N1(k−1)(x,y)+αk,14(x)×(∂k(S1(4r)−M1(2r))∂xk(x1,y)−∂kN1(k−1)∂xk(x1,y))−βk,14(x)∂kN1(k−1)∂xk(0,y),k=1,2,…  ((x,y)∈H1).

(ii) Denote

(113) αk,21(x)=(x−x2)kk!(1−x1−x2)k+1,βk,21(x)=(x−1)kk!(x2−xx2−1)k+1, k=0,1,….

Define *M*_2_^(*τ*_*r*_)^(*x*, *y*) by induction as follows:

(114) M2(0)(x,y)=S1(lr)(x2,y)α0,21(x),M2(k)(x,y)=M2(k−1)(x,y)+αk,21(x)×(∂kS1(lr)∂xk(x2,y)−∂kM2(k−1)∂xk(x2,y))−βk,21(x)∂kM2(k−1)∂xk(1,y),k=1,2,… ((x,y)∈H2).

Denote

(115) αk,22(y)=(y−y2)kk!(yy2)k+1,βk,22(y)=ykk!(y−y2−y2)k+1, k=0,1,….

Define *N*_2_^(*r*)^(*x*, *y*) by induction as follows:

(116) N2(0)(x,y)=(S2(lr)−M2(τr))(x,y2)α0,22(y),N2(k)(x,y)=N2(k−1)(x,y)+αk,22(y)×(∂k(S2(lr)−M2(τr))∂yk(x,y2)−∂kN2(k−1)∂yk(x,y2))−βk,22(y)∂kN2(k−1)∂yk(x,0),k=1,2,… ((x,y)∈H2).

(iii) Denote

(117) αk,31(y)=(y−y3)kk!(1−y1−y3)k+1,βk,31(y)=(y−1)kk!(y3−yy3−1)k+1, k=0,1,….

Define *M*_3_^(*k*)^(*x*, *y*) by induction as follows:

(118) M3(0)(x,y)=S2(lr)(x,y3)  α0,31(y).M3(k)(x,y)=M3(k−1)(x,y)+αk,31(y)×(∂kS2(lr)∂yk(x,y3)−∂kM3(k−1)∂yk(x,y3))−βk,31(y)∂kM3(k−1)∂yk(x,1),k=1,2,… ((x,y)∈H3).

Denote

(119) αk,32(x)=(x−x3)kk!(1−x1−x3)k+1,βk,32(x)=(x−1)kk!(x3−xx3−1)k+1, k=0,1,….

Define *N*_3_^(*k*)^(*x*, *y*) by induction as follows:

(120) N3(0)(x,y)=(S3(lr)−M3(τr))(x3,y)α0,32(x),  N3(k)(x,y)=N3(k−1)(x,y)+αk,32(x)×(∂k(S3(lr)−M3(τr))∂yk(x3,y)−∂kN3(k−1)∂xk(x3,y))−βk,32(x)∂kN3(k−1)∂xk(1,y),k=1,2,… ((x,y)∈H3).

(iv) Denote

(121) αk,41(x)=(x−x4)kk!(xx4)k+1,  βk,41(x)=(x−x4)kk!(x−x4)k+1, k=0,1,….

Define *M*_4_^(*k*)^(*x*, *y*) by induction as follows:

(122) M4(0)(x,y)=S3(lr)(x4,y)α0,41(x),M4(k)(x,y)=M4(k−1)(x,y)+αk,41(x)×(∂kS3(lr)∂xk(x4,y)−∂kM4(k−1)∂xk(x4,y))−βk,41(x,y)∂kM4(k−1)∂xk(0,y),k=1,2,… ((x,y)∈H4).

Denote

(123) αk,42(y)=(y−y4)kk!(1−y1−y4)k+1,βk,42(y)=(y−1)kk!(y4−yy4−1)k+1, k=0,1,….

Define *N*_4_^(*k*)^(*x*, *y*) by induction as follows:

(124) N4(0)(x,y)=(S4(lr)−M4(τr))(x,y4)α0,42(y),N4(k)(x,y)=N4(k−1)(x,y)+αk,42(y)×(∂k(S4(lr)−M4(τr))∂yk(x,y4)−∂kN4(k−1)∂yk(x,y4))−βk,42(x,y)∂kN4(k−1)∂xk(x,1),k=1,2,… ((x,y)∈H4).

## 5. Corollaries

By using the extension method given in Section 3, we discuss the two important special cases.

### 5.1. Smooth Extensions of Functions on a Kind of Domains

Let *Ω* be a trapezoid with two curved sides:

(125) $$\begin{array}{c} \Omega =\left\{\left(x,y\right):x_{1}\leq x\leq x_{2},\;\;\;\eta \left(x\right)\leq y\leq \xi \left(x\right)\right\}, \end{array}$$

where *L*_1_ < *η*(*x*) < *ξ*(*x*) < *L*_2_ (*x*_1_ ≤ *x* ≤ *x*_2_), *η*, *ξ* ∈ *C* ^*m*^([*x*_1_, *x*_2_]). Denote the rectangle *D* = [*x*_1_, *x*_2_]×[*L*_1_, *L*_2_]. Then *D* = *G*_1_⋃*Ω*⋃*G*_2_, where *G*_1_ and *G*_2_ are both trapezoids with a curved side:

(126) $$\begin{array}{c} G_{1}=\left\{\left(x,y\right):x_{1}\leq x\leq x_{2},\;\;L_{1}\leq y\leq \eta \left(x\right)\right\},\\ G_{2}=\left\{\left(x,y\right):x_{1}\leq x\leq x_{2},\;\;\xi \left(x\right)\leq y\leq L_{2}\right\}. \end{array}$$

Suppose that *f* ∈ *C* ^*q*^(*Ω*) (*q* is a nonnegative integer). We will smoothly extend *f* from *Ω* to the trapezoids *G*_1_ and *G*_2_ with a curved side, respectively, as in Section 3.2, such that the extension function *F* is smooth on the rectangle *D*. Moreover, we will give a precise formula. It shows that the index of smoothness of *F* depends on not only smoothness of *f* but also smoothness of *η*, *ξ*.

Denote *a*_0,1_(*x*, *y*) = (*y* − *L*_1_)/(*η*(*x*) − *L*_1_) and

(127) ak,1(x,y)=(y−η(x))kk!(y−L1η(x)−L1)k+1,k=1,2,…  (x1≤x≤x2,y∈ℝ).

We define {*S*_1_^(*k*)^(*x*, *y*)} on *G*_1_ as follows. Let

(128) $$\begin{array}{c} S_{1}^{\left(0\right)}\left(x,y\right)=f\left(x,\eta \left(x\right)\right)a_{1,0}\left(x,y\right)\;\left(\left(x,y\right)\in G_{1}\right), \end{array}$$

and let *k*_0_ be the maximal integer satisfying 1 + 2 + ⋯+*k*_0_ ≤ *q*. For *k* = 1,2,…, *k*_0_, we define

(129) S1(k)(x,y)=S1(k−1)(x,y)+a1,k(x,y)×(∂kf∂yk(x,η(x))−∂kS1(k−1)∂yk(x,η(x))).

Then *S*_1_^(*k*)^ ∈ *C* ^*λ*_*k*_^(*G*_1_), where *λ*_*k*_ = min⁡{*q* − 1 − 2 − ⋯−*k*, *m*}.

Denote   *a*_0,2_(*x*, *y*) = (*L*_2_ − *y*)/(*L*_2_ − *ξ*(*x*)) and

(130) ak,2(x,y)=(y−ξ(x))kk!(L2−yL2−ξ(x))k+1,k=1,2,… (x1≤x≤x2,y∈ℝ).

We define {*S*_2_^(*k*)^(*x*, *y*)} on *G*_2_ as follows. Let

(131) $$\begin{array}{c} S_{2}^{\left(0\right)}\left(x,y\right)=f\left(x,\xi \left(x\right)\right)a_{0,2}\left(x,y\right)\;\left(\left(x,y\right)\in G_{2}\right). \end{array}$$

For *k* = 1,2,…, *k*_0_, define

(132) S2(k)(x,y)=S2(k−1)(x,y)+ak,2(x,y)×(∂kf∂yk(x,ξ(x))−∂kS2(k−1)∂yk(x,ξ(x))), ((x,y)∈G2).

Then *S*_2_^(*k*)^ ∈ *C* ^*λ*_*k*_^(*G*_2_), where *λ*_*k*_ is stated as above.

An argument similar to Lemmas 5 and 6 shows that, for 0 ≤ *k* ≤ *k*_0_ and 0 ≤ *i* + *j* ≤ min⁡{*k*, *λ*_*k*_},

(133) ∂i+jS1(k)∂xi∂yj(x,η(x))=∂i+jf∂xi∂yj(x,η(x)),  ∂i+jS2(k)∂xi∂yj(x,ξ(x))=∂i+jf∂xi∂yj(x,ξ(x)),(x1≤x≤x2).

A direct calculation shows that the number

(134) $$\begin{array}{c} \tau \left(q,m\right)=\min\left\{\left[\sqrt{2q+\frac{9}{4}}-\frac{3}{2}\right],m\right\} \end{array}$$

is the maximal value of integers *k* satisfying *k* ≤ *λ*_*k*_, where [·] expresses the integral part. So *τ*(*q*, *m*) ≤ *λ*_*τ*(*q*,*m*)_.

By (133), we get that, for 0 ≤ *i* + *j* ≤ *τ*(*q*, *m*),

(135) ∂i+jS1(τ(q,m))∂xi∂yj(x,η(x))=∂i+jf∂xi∂yj(x,ξ(x)),∂i+jS2(τ(q,m))∂xi∂yj(x,ξ(x))=∂i+jf∂xi∂yj(x,ξ(x)),(x1≤x≤x2).

Note that

(136) S1(τ(q,m))∈Cλτ(q,m)(G1),  S2(τ(q,m))∈Cλ(q,m)(G2),τ(q,m)≤λτ(q,m)≤q,

and the assumption *f* ∈ *C* ^*q*^(*Ω*). Now we define a function on *D* by

(137) $$\begin{array}{c} F_{q,m}\left(x,y\right)=\{\begin{array}{cc} f\left(x,y\right), & \left(x,y\right)\in \Omega ,\\ S_{1}^{\left(\tau (q,m)\right)}\left(x,y\right), & \left(x,y\right)\in G_{1},\\ S_{2}^{\left(\tau (q,m)\right)}\left(x,y\right), & \left(x,y\right)\in G_{2}. \end{array} \end{array}$$

From this and (135), we have *F*_*q*,*m*_ ∈ *C* ^*τ*(*q*,*m*)^(*D*). This implies the following theorem.

**Theorem 12**

Let the domain *Ω* and the rectangle *D* be stated as above. If *f* ∈ *C* ^*q*^(*Ω*), then the function *F*_*q*,*m*_(*x*, *y*), defined in (137), is a smooth extension of *f* from *Ω* to *D* and *F*_*q*,*m*_ ∈ *C* ^*τ*(*q*,*m*)^(*D*), where *τ*(*q*, *m*) is stated in (134).

Especially, for *q* = 0 and *m* ≥ 0, we have *τ*(*q*, *m*) = 0, and so *F*_0,*m*_ ∈ *C*(*D*); for *q* = 2 and *m* ≥ 1, we have *τ*(*q*, *m*) = 1, and so *F*_2,1_ ∈ *C* ^1^(*D*); for *q* = 5 and *m* ≥ 2, we have *τ*(*q*, *m*) = 2, and so *F*_5,2_ ∈ *C* ^2^(*D*).

### 5.2. Smooth Extensions of Univariate Functions on Closed Intervals

Let *f* ∈ *C* ^*q*^([*x*_1_, *x*_2_]) and [*x*_1_, *x*_2_]⊂(0,1). In order to extend smoothly *f* from [*x*_1_, *x*_2_] to [0, *x*_1_], we construct two polynomials

(138) a0(k)(x)=(x−x1)kk!(xx1)k+1,b0(k)(x)=xkk!(x1−xx1)k+1, k=0,1,….

Define *S*_0_^(0)^(*x*) = *f*(*x*_1_)(*x*/*x*_1_) and for *k* = 1,…, *q*,

(139) S0(k)(x)=S0(k−1)(x)−a0(k)(x)(f(k)(x1)−(S0(k−1))(k)(x1))−b0(k)(x)(S0(k−1))(k)(0) (0≤x≤x1).

Then *S*_0_^(*q*)^(*x*) is a polynomial of degree ≤2*q* + 1.

Similar to the proof of Lemma 5, we get

(140) (S0(q))(k)(0)=0,  (S0(q))(k)(x1)=f(k)(x1),k=0,1,…,q.

It is also easy to check directly them.

Again extend smoothly *f* from [*x*_1_, *x*_2_] to [*x*_2_, 1], we construct two polynomials

(141) a1(k)(x)=(x−x2)kk!(1−x1−x2)k+1,b1(k)(x)=(x−1)kk!(x2−xx2−1)k+1, k=0,1,….

Define   *S*_1_^(0)^(*x*) = *f*(*x*_2_)((1 − *x*)/(1 − *x*_2_)) and for *k* = 1,…, *q*,

(142) S1(k)(x)=S1(k−1)(x)−a1(k)(x)(f(k)(x2)−(S1(k−1))(k)(x2))−b1(k)(x)(S1(k−1))(k)(1) (x2≤x≤1).

Then *S*_1_^(*q*)^(*x*) is a polynomial of degree ≤2*q* + 1.

Similar to the proof of Lemma 5, we get

(143) (S1(q))(k)(x2)=f(k)(x2),(S1(q))(k)(1)=0 (k=0,1,…,q).

Therefore, we obtain the smooth extension *F* from [*x*_1_, *x*_2_] to [0,1] by

(144) $$\begin{array}{c} F\left(x\right)=\{\begin{array}{cc} f\left(x\right), & x\in \left[x_{1},x_{2}\right],\\ S_{0}^{\left(q\right)}\left(x\right), & x\in \left[0,x_{1}\right],\\ S_{1}^{\left(q\right)}\left(x\right), & x\in \left[x_{2},1\right], \end{array} \end{array}$$

where *S*_0_^(*q*)^(*x*) and *S*_1_^(*q*)^(*x*) are polynomials of degree 2*q* + 1 defined as above, and *F* ∈ *C* ^*q*^([0,1]) and *F* ^(*l*)^(0) = *F* ^(*l*)^(1) = 0 (*l* = 0,1, ..., *q*). From this, we get the following.

**Theorem 13**

Let *f* ∈ *C* ^*q*^([*x*_1_, *x*_2_]) and [*x*_1_, *x*_2_]⊂(0,1). Then there exists a function *F* ∈ *C* ^*q*^([0,1]) satisfying *F*(*x*) = *f*(*x*) (*x*_1_ ≤ *x* ≤ *x*_2_) and *F* ^(*l*)^(0) = *F* ^(*l*)^(1) = 0 (*l* = 0,1,…, *q*).

Let *f* ∈ *C* ^*q*^([*x*_1_, *x*_2_]) and [*x*_1_, *x*_2_]⊂(0,1), and let *F* be the smooth extension of *f* from [*x*_1_, *x*_2_] to [0,1] which is stated as in Theorem 12. Let *F*_*p*_ be the 1-periodic extension satisfying *F*_*p*_(*x* + *n*) = *F*(*x*) (0 ≤ *x* ≤ 1, *n* ∈ *ℤ*). Then *F*_*p*_ ∈ *C* ^*q*^(ℝ) and *F*_*p*_(*x*) = *f*(*x*) (*x* ∈ [*x*_1_, *x*_2_]). We expand *F*(*x*) into the Fourier series which converges fast. From this, we get trigonometric approximation of *f* ∈ *C* ^*q*^([*x*_1_, *x*_2_]). We also may do odd extension or even extension of *F* from [0,1] to [−1,1], and then doing periodic extension, we get the odd periodic extension *F*_*p*_^*o*^ ∈ *C* ^*q*^(ℝ) or the even periodic extension *F*_*p*_^*e*^ ∈ *C* ^*q*^(ℝ). We expand *F*_*p*_^*o*^ or *F*_*p*_^*e*^ into the sine series and the cosine series, respectively. From this, we get the sine polynomial approximation and the cosine polynomial approximation of *f* on [*x*_1_, *x*_2_]. For *F* ∈ *C* ^*q*^(*x*) (*x* ∈ [0,1]), we pad zero in the outside of [0,1] and then the obtained function *F* ^*c*^ ∈ *C* ^*q*^(ℝ). We expand *F* ^*c*^ into a wavelet series which converges fast. By the moment theorem, a lot of wavelet coefficients are equal to zero. From this, we get wavelet approximation of *f* ∈ *C* ^*q*^([*x*_1_, *x*_2_]).
